# Factors influencing women’s access to the maternity waiting home in rural Southwest Ethiopia: a qualitative exploration

**DOI:** 10.1186/s12884-020-02988-8

**Published:** 2020-05-14

**Authors:** Kindie Mitiku Kebede, Kebadnew Mulatu Mihrete

**Affiliations:** 1 Department of Public Health, College of Health Sciences, Mizan -Tepi University, PO. Box 260, Tepi, Ethiopia; 2 Departments of Epidemiology and Biostatics, Bahir Dar University, Bahir Dar, Ethiopia

**Keywords:** Maternity waiting home, Utilization, Access, And rural Southwest Ethiopia

## Abstract

**Background:**

Maternity Waiting Homes (MWHs) have been advocated to improve the utilization of skilled birth attendants. Nevertheless, delivery attended by skilled personnel is low in Ethiopia and may indicate that the utilization of MWH is also low. The aim of this study is to explore the factors influencing women’s access to the MWHs in rural Southwest Ethiopia.

**Methods:**

Qualitative data were collected through focus group discussions with MWHs users and in-depth interviews with MWHs non-users, health extension workers and the clinicians. Four focus group discussions and 18 in-depth interviews were conducted between May 1 and June 1, 2017. Furthermore, observations were made to assess the availability of basic facilities at selected MWHs. Data were thematically analyzed using NVivo version 7. The concept of access defined by Thiede et al was applied to guide the analysis.

**Results:**

Women had interest on MWHs and are aware of the existence of MWHs in their immediate vicinity. Health information disseminations and referral linkages by frontline health workers enabled women to timely access the MWHs. However, Women didn’t understand the aims and benefits of MWHs. At the facility level, there were attempts to improve the acceptability of MWHs by allowing women to choose their delivery positions. But, participants claimed lack of privacy and presence of disrespectful care. Physical barriers (long distance, unavailability of transport options & unfavorable roads) were considered as potential problems for women residing in remote areas. MWH users mentioned absences of sufficient basic facilities, poor quality and varieties of food. Because of insufficient facilities, the cost of living was high for most users. The communities try to overcome the indirect costs through contributions in-kind and in-cash.

**Conclusions:**

The factors influencing women’s access to the MWHs were structural and individual and resonate with Thiede et al. dimensions of access. A better understanding of which factors are most influential in preventing women’s access to the MWHs in rural Southwest Ethiopia is needed to appropriately target interventions.

## Background

According to the world health organization, a MWH is defined as a “residential facility located near a qualified medical facility where women can await their delivery and be transferred shortly before delivery or earlier should a complication arise” [1].

MWH has numerous advantages [2–5]. It increases the use of skilled birth attendants [2–4], decrease maternal mortality [2, 4] and prevents adverse pregnancy outcomes [5]. Despite these benefits, its utilization is low in sub-Saharan African countries [6–8] and a recent systematic review showed that there are several factors that influence the utilization of MWHs [9].

Like other maternal health care services, utilization of MWH is deterred by long distance [8–10], high cost of transportation [11], lack of transport options, unfavorable road conditions [7] and poor awareness about the benefits of MWHs [8, 12]. Available evidence showed that MWHs have poor infrastructures and lack basic facilities that are deemed vital for women and their visiting families [9, 13]. In this regard, unavailability of food at the MWHs was cited as a major barrier in many countries implementing the MWH program [7, 10, 14].

Access to the MWH is also affected by lack of decision-making power [8, 10, 13] and poor quality of MWH as well as delivery care services [11, 13]. Moreover, provision of culturally inappropriate care may also discourage the acceptability of MWHs. For instance, a recent qualitative synthesis confirmed the presence of culturally inappropriate care in the MWHs of low and middle-income countries that deterred women’s ability to use MWHs [9].

In Ethiopia, the practice of MWH spans more than three decades. The first MWH was established at Atat hospital, in Southern regions of Ethiopia in 1985 [14]. Despite a long period of implementation, utilization of skilled birth attendant is still low in Ethiopia. According to the 2016 Mini Ethiopian Demographic and Health Survey, the majority of women (85%) did not give birth at health facilities [15].

The Federal Ministry of Health’s (FMOH’s) has planned to reduce the maternal mortality ratio (MMR) to 199 deaths per 100,000 live births and to increase skilled birth attendance (SBA) to 90% by 2020 [16]. To achieve these ambitious plans, maternal health care services need to be accessible for women residing in rural areas. The MWHs have been advocated by the government to improve women’s ability to access the health facilities for delivery [13, 14].

Women are often advised to go to the MWHs during their last weeks of pregnancy. Then, they wait at the MWHs until labour starts. Nurses perform an initial evaluation for pregnant women that are admitted to the MWHs. During the initial visit, women are expected to get physical examinations & blood pressure measurements. Nurses are also expected to provide health education and promotion services in a regular bases. Women are also linked with other maternal health services such as antenatal care (ANC), immunization and HIV counseling and testing services. During their stay in the MWHs, women are not expected to pay for the maternal health services received from the MWHs [14].

Though MWH program is currently being implemented in this study area, the proportion of births attended by skilled birth attendants is low. A recent study in Sheka zone of this study area showed that only 25.1% of women gave birth at health facilities [17]. The proportion of births attended by skilled personnel is even lower in rural areas of this region. For instance, only 16.7% of rural women in Benchi-Maji zone of this study area gave birth at health facilities where there are skilled birth attendants in 2017 [18].

The low proportion of births attended by skilled birth attendants in this study area may suggest that the MWH is not accessed by women. Nevertheless, there is a scarcity of studies on factors influencing women’s access to the MWHs in rural southwest Ethiopia. The available studies in Ethiopia are nationwide [13, 14] and conducted on women’s intention to use MWHs [12]. To investigator’s knowledge, this study is the first qualitative study on the factors influencing women’s access to the MWH in rural Southwest Ethiopia. Hence, this study was conducted to get a deeper understanding of the factors influencing women’s access to the MWH through the lens of women, health extension workers and other health professionals (clinicians) in rural Southwest Ethiopia.

### Guiding framework

To guide the analysis, we used the ‘A-frame’ of access proposed by Thiede et al. [19] where they observed the concept of access in both individual and health system directions. According to Thiede et al., access is defined in three dimensions; availability (physical access), affordability (financial access) and acceptability (cultural access). Availability of information cuts across all three dimensions of access. The definition of each dimension of access is found as an additional file (Additional file 1). Studies conducted on the factors influencing women’s access to the MWHs using the ‘A-frame work’ of access are scare in Ethiopia. Particularly, in rural south west Ethiopia, there is no study that explored the factors influencing women’s access to the MWHs using the ‘A-frame work’ of access. To appropriately target interventions, the factors influencing women’s access to the MWHs needs to be looked at the individual and health system directions. Therefore, this study aims to explore the factors influencing women’s access to the MWHs by applying the ‘A-frame work’ of access proposed by Thiede et al.

## Methods

### Setting

This study was conducted in selected catchment areas with MWHs in Kaffa, Sheka, and Benchi-Maji zone in Southwest Ethiopia between May 1 and June 1, 2017. A catchment area refers to a geographical area constructed around a clinic, describing the population that uses its services [20]. In this study context, catchment area refers to the area constructed around the MWHs, describing the population that uses this service. Since MWHs in Ethiopia and in this study sites are constructed around the health centers or hospitals, the catchment area of MWHs is similar to the catchment area of the health centers or hospitals. The total projected population of Keffa, Sheka, and Benchi-Maji zone was 2258, 803, 258,398 and 838,235 respectively in 2017. Women of reproductive age in all zones constituted approximately 23% of the population [21–23]. The health center in each zone provides both preventive and curative services including maternal health care services. More complicated cases are usually referred to Tepi General Hospital, Gebre Tsadiq Shawo Memorial hospital, Mizan-Tepi University Teaching, and Referral Hospital that can perform emergency surgical procedures and provide blood transfusions as well as other specialized services.

#### Study design

A qualitative approach using focus groups and in-depth interviews was employed. The rationale for this approach is to explore more in-depth about factors influencing women’s access to the MWHs. In addition, observations of MWHs using checklists adopted from a standardized protocol for managing the MWHs in Ethiopia were used to assess the availability of basic facilities.

The checklists used for observation of selected MWHs are found as supplementary file (Additional file 2). Field notes were also taken during observation.

The participants partook in the interviews were: 1) MWHs users, 2) MWHs non-users, 3) health extension (HEWs) and 4) clinicians. The FGDs were conducted with MWHs users and the IDIs were carried out with MWHs non-users, HEWs and clinicians. All of the participants were selected using purposive sampling technique.

#### Sample size

The sample size was determined based on the saturation of data. We considered data saturation when new ideas are no longer obtained from the interviews. Thus, a total of 4 FGDs and 18 IDIs (5 with clinicians, 6 with HEWs and 7 with MWH non-users) were conducted. The FGDs were conducted with groups of 5–9 MWH users identified by the principal investigators (KMK and KMM).

#### Inclusion criteria

Predetermined inclusion criteria were used to recruit all participants. Four MWH sites (Kite MWH, Sheko MWH, Andracha MWH, and Chena MWH) that had sufficient numbers of MWH users for FGDs were selected. In addition to these four MWH sites, we selected two other MWH sites (Kubito MWH and Shey Benchi MWH) for in-depth interviews. We added these two extra MWHs for IDIs because we were informed that women were not using these MWHs frequently. MWH users who were admitted to the MWHs and stayed at least 1 week were purposefully selected by the principal investigator with the consultation of clinical staffs who know very well and supervised them. MWH users were carefully chosen to capture the opinions of different age groups and educational backgrounds.

MWH non-users that had a child less than 6 months were selected to minimize recall bias and to identify recent factors influencing women’s access to the MWHs. HEWs helped us to identify non-users that had a child less than 6 months in the selected areas. Like MWH users, MWH non-users were also carefully selected to capture the opinion of different age groups and educational backgrounds. Frontline health workers (clinical staffs and HEWs) were purposely selected. We purposely selected these health workers because they have frequent contact with women and responsible for community mobilization, identification, and referral of women to the MWHs. HEWs and clinical staffs that had at least 2 years experience were eligible for in-depth interviews. Finally, eight MWHs that were functional for the last 1 year preceding the study (Kite MWH, Sheko MWH, Andracha MWH, Chena MWH, Kubito MWH, Shey Benchi MWH, Shishonde MWH, and Bear MWH) were observed to assess the availability of infrastructures & basic facilities.

#### Data collection procedures and tools

Piloted interview guiding questions organized according to the ‘A-frame’ of access proposed by Thiede et al were used for both FGDs and IDIs. An additional file 3 shows the guiding questions (additional file 3). All of the participants were approached face-to-face and none of them refused to participate. The FGDs took place at the MWHs in spaces where no other individuals presented to ensure their privacy. IDIs with MWH non-users were conducted in their homes in the absence of other individuals. In-depth interviews and FGDs with women were conducted in the local language of Benchigna, Shekinano, and Kafinano. The in-depth interviews with health workers were conducted with “Amharic” language.

Interviews with health workers were conducted by principal investigators (KMK & KMM). One of the principal investigators recorded the interview (KMK) and the other facilitated the interview (KMM).

Two female research assistances conducted the interviews with MWH users and non-users; one acted as recorder and the other as a facilitator. All interviews lasted between 45 min to 1 h and were audio-recorded.

### Research team composition and their relationship with participants

The data collection team was composed of two male primary investigators (KMK & KMM) and two research assistants, all of whom had tertiary level qualifications. Both the research assistants were female. The investigators were university lecturers and public health specialists. The research assistances were BSc holders in public health and trained before the actual data collection period. All of the research assistants did not have a relationship with the participants before the commencement of the study. However, participants were informed about the reasons for doing the research in the study area.

#### Data analysis

Qualitative data generated from FGDs and IDIs were transcribed verbatim and translated into English. The hard copy notes taken during observation of MWHs were converted to soft copy. Then, the translated data were entered into NVivo version 7. Thematic content analysis of the data was performed using ‘A-frame’ of access proposed by Thiede et al [19]. Thus, our findings were compared with existing theory [24].

Generally, the data analysis process involved four steps. First, transcripts were read by each author (KMK and KMM) for general impression. Second, each author prepared a codebook using one transcript from each group of participants. Third, both investigators jointly made a further revision to the codebook.

Any disagreement was resolved by consensus. Using this code book as a guiding tool, both authors completed the coding process separately using NVivo version 7.

Fourth, the codes were sorted into preliminary categories using the ‘A-frame’ of access proposed by Thiede and colleagues by both authors. Emerging codes were examined to see how well they fit with themes from the ‘A-frame’ of access. The themes were also compared across different groups of participants to check the ranges of and similarities of the participants’ views. Furthermore, themes were checked to ascertain that they had not been over or under-represented.

To improve the trustworthiness of the results, some HEWs and clinicians were invited to comment on the research findings and themes during presentation of preliminary finding at Mizan-tepi University. The Consolidated Criteria for Reporting Qualitative Research (COREQ) checklist was used to guide reporting of this study [25]. The COREQ checklist contains 32 items broadly categorized into 3 domains:1) research team and reflexivity, 2) study design & 3) data analysis & reporting. The page numbers where each of the items listed in this checklist are reported as an additional file (Additional file 4). Narrative texts followed by participants’ quotations were applied around the themes to illustrate the themes.

#### Ethical considerations

Ethical clearance was obtained from Mizan-Tepi University research directorate. The aim and potential benefits of the study were discussed with all of the participants. Before enrolling participants in any of the interviews, informed oral consent was taken. Oral consent was taken because majority of the women participated in this study were illiterate. The verbal consent was approved by the ethics committee.

## Results

The background characteristics of the study participants are described in Table 1. As shown in Table 1, all women interviewed had no more than secondary level of education. A gap between antenatal care (ANC) utilization and health facility delivery was also observed in which all women attended ANC but, few gave birth to their young child at health facilities (Table 1).

**Table 1:** Background characteristics of MWH users and non-users in rural Southwest Ethiopia, 2017

Characteristics		MWH users (N)	MWH non-users (N)
Age	17–19	9	0
	20–24	6	5
	25–29	8	2
	30–34	2	0
	≥35	0	0
Marital status	Married	25	7
	Not married	0	0
Education status	No formal education	14	6
	Primary(1–8)	10	1
	Secondary(9–12)	1	0
	Tertiary(> 12)	0	0
Sex	Female	25	7
	Male	0	0
Occupation	Farming	18	5
	Government employed	0	0
	Housewife	6	2
	Others	1	0
Number of children	Had no children	6	1
	1	7	1
	2–4	9	5
	≥5	3	0
ANC	Yes	25	7
	No	0	0
Number of ANC	1	2	1
	1–4	15	2
	≥4	8	4
Place of delivery (the youngest child)	Home	15	5
	Health facility	8	2

### Availability of infrastructures and basic facilities

The availability of infrastructures and basic facilities among selected MWHs is described in Table 2. All of the MWHs were found in the health facilities. However, many of them didn’t have sufficient classes to accommodate pregnant women. Four of the eight observed MWHs had only one class & only four of the MWHs had pipe water. Moreover, seven of the eight observed MWHs were made up of corrugated iron (Table 2).

**Table 2:** The availability of infrastructures and basic facilities among selected MWHs in rural Southwest Ethiopia, 2017

Variables		Frequency
MWH manual	Yes	3
	No	5
Found inside in the health center	Yes	8
	No	0
Numbers of classes	1	4
	2–4	3
	≥5	1
Type of house	Traditional huts	1
	Corrugated iron	7
Kitchen	Yes	6
	No	2
Latrine	Yes	6
	No	2
Pipe water	Yes	4
	No	4
Electricity	Yes	7
	No	1
Shower	Yes	2
	No	6
Television	Yes	2
	No	6
Registration book	Yes	8
	No	0
Have gateway	Yes	8
	No	0
The gateway can pass a car	Yes	7
	No	1
Poster about danger signs	Yes	1
	No	7

### Factors influencing women’s access to the MWHs

The factors identified by MWH users, MWH non-users, HEWs and clinicians are grouped according to the dimensions of access as illustrated in Table 3 and discussed in detail below.

**Table 3:** The coding structures organized according to the dimensions of access in rural Southwest Ethiopia, 2017

Themes	Participants				
	MWH non-users	MWH users	HEWs	Clinicians	Observation
Acceptability	Women’s interest to stay in the MWHs Community interest to use MWH Women not allowed to practice their culture at MWHs	Community interest to use MWHs Women’s interest to stay in the MWHs	Community interest to use MWHs Lack of privacy The absence of respect	Community interest to use MWHs Women to choose a delivery position Presence of separate class for ethnic minorities	
Availability	Unavailability of food Unavailability of water Lack of transport options Long distance Unavailability of a network to call an ambulance Lack of support	The absence of recreation mechanisms Narrow classes The absence of varieties of food Unavailability of water Unavailability of food Lack of transport options Long distance Unfavorable road condition Lack of support	The absence of sufficient matters Unavailability of food Unavailability of water Poor quality of food The absence of varieties of food Food hasn’t preferred by women Shortage of utensils Lack of transport options Long distance Lack of support	Narrow classes Families not allowed to stay at MWH Unavailability of food The absence of varieties of food Food hasn’t preferred by women Shortage of utensils Unavailability of water Lack of transport options Long distance Lack of support	Narrow classes The absence utensils No separated latrine The absence of sufficient classes Unavailability of water
Affordability	Community contribution in kind Community contribution in cash	Indirect payments Community contribution in kind	Community contribution in kind Community contribution in cash	Community contribution in kind Community contribution in cash	Affordability
Information	HEWs as a source of information about MWHs Misinformation when to go to MWHs Awareness about the consequences of home delivery Awareness about the existence of MWHs	HEWs as a source of information about MWHs Awareness about the existence of MWHs Awareness about the consequences of home delivery Limited knowledge about the aims and benefits of MWHs Education and counseling at MWH	HEWs as a promoter of referral linkages HEWs as a source of information about MWH Awareness about the existence of MWHs Limited knowledge about the aims and benefits of MWHs Belief on MWHs users will have an operation Perception on MWH reserved for weak women	HEW as a promoter of referral linkages Limited knowledge about the aim of MWH Awareness about the consequences of home delivery Limited knowledge about the aims and benefits of MWHs Education and counseling at MWH	

### Acceptability

MWHs were constructed by the government in collaboration with the communities. Involving the communities during construction of MWHs enabled them to develop ownership of the MWHs. The clinicians and HEWs participated in the IDIs highlighted that the communities and pregnant women had interest to use MWHs. Clinicians also mentioned that they strived a lot to increase the acceptability of MWHs by pregnant women and communities at large. For instance, an experienced health workers participated in the in-depth interview mentioned that they allow women to choose their preferred delivery positions.

*“In this community, women don’t want to give birth in lying down position so they don’t want to stay on the delivery beds. They believe that staying on the delivery beds delay the laboring process. They prefer to give birth on the ground than staying on the delivery beds. In fact, we allow them to choose their own delivery position.”* (Clinician, IDI)

However, MWHs non-users revealed that there were instances in which women were not allowed to practice their traditions in the MWHs.

*“If we give birth at home, we will prepare the traditional medicine. We put a stone over the fire. When the stone becomes hot, we put it in the bucket that contains water. The leaf that is considered as medicine will be added over the stone. Then, women wash their body with the warm water. They believe that this clean body; heal wounds and dry blood. If we go to the clinics, we will not get this traditional medicine.”* (MWH non-user woman, IDI)

HEWs also shared their concerns regarding to women’s expectation from the health workers. For example, women expect respect and privacy from health care providers. However, there were instances in which the health workers may not respect and keep the privacy of women.

*“Before, we were referring more than 20 women per month. Recently, we are not referring pregnant women like before. This is because the MWH is not accommodating women and the health professionals are not giving respectful care.”* (HEW, IDI)

### Availability

Women living in rural and most remote areas faced physical barriers to timely accessing the MWHs located in urban areas. Access to the MWHs was difficult because of unfavorable road conditions and long distance between the MWHs and women’s home. Traveling long distance was particularly a critical challenge for women residing in most remote and mountainous zones of southwest Ethiopia. Some women may even walk several miles to reach the MWHs.

*“I came on foot. It took around 7 hours to reach the MWHs. Because of fear of traveling such a long distance, women don’t decide to come to the MWHs. So, some women deliver at home and suffer from a retained placenta. Since there is no favorable road, the ambulances will not come.” (*MWH user, FGD)

In addition to long distance and unfavorable roads, women’s ability to reaching the MWHs was constrained by unavailability of transport options. Ambulances were the only option for those women living in remote areas. However, pregnant women were not using the ambulance services because of two reasons; first) most of the time ambulances were reserved for only emergency cases; second) the condition of the road, particularly, in the rainy season was not suitable for the ambulances.

*“In our kebele, during the rainy season, the road becomes muddy. So the ambulances don’t come. Many women suffered from retained placenta. Instead of coming to the health center, they search traditional medicine.” (*MWH users, FGD)

Poor MWHs infrastructures and unavailability of basic facilities such as food, water, utensils and recreation mechanisms has been cited as major barriers for women to accessing the MWHs. The available waiting homes were insufficient and the classes were narrow to accommodate a numbers of women at the same time. Because of insufficient classes, women were not allowed to bring their families or there may be a restriction of the numbers of families to stay with pregnant women in the MWHs.

*“The classes are crowded. If we mobilize and many mothers come to the MWHs, where do they stay? Where do they prepare food?”* (Clinician, IDI)

*“The waiting classes are narrow. Because of this, sometimes, we don’t allow family members to stay* with pregnant women *in the MWHs. This disappoints family members.”* (Clinician, IDI)

Unavailability of food was a serious challenge for most MWHs users. The availability of food varied from one MWH to the other. Some MWHs provided food in regular bases others not. All women felt that unavailability of food was one of the main reasons why women don’t come & stay in the MWHs. MWH users also condemned the quality and varieties of food. The foods served in some MWHs were not even culturally preferred by women**.**

*“During pregnancy, we need varieties of foods. If we don’t get these foods, we will not be well. If I were at my home, my husband would buy the food I preferred. But, once we came here, there is nobody who buys what we want. Here in the MWH, there is nothing except oil and bread.” (*MWH user, FGD)

*“In the MWH, they only provide flour for preparing bread & porridge. However, Women don’t have interest to eat bread & porridge. They prefer to consume local foods, for example, ‘Godere’ and ‘Chemo’ than the foods provided at the MWHs.”* (HEWs, IDIs)

Moreover, MWH users and health workers mentioned that lack of personnel that could provide support for women and their children left at home discouraged some pregnant women to timely accessing the MWHs. MWH users acknowledged their husbands support during pregnancy. Some husbands encouraged their wife to stay in the MWHs by bringing food and even accompanying them during referrals. But, as noted by other MWH users, husbands can also prevent women from utilizing the MWHs.

*“There is nobody who would take good care for their young children if women come and stay at the maternity waiting home. Because of this, some women give birth at their home.”* (MWH user, FGD)

*“There are some husbands who don’t allow their wife staying in the MWHs. Some husbands even don’t hear what the HEWs told them. This is because they live in rural and they don’t know the benefits of MWHs*.” (MWH user, FGD)

### Affordability

Maternal health services in Ethiopia are free. So, pregnant women are not expected to pay for the services received from the MWHs. Nevertheless, MWH users felt that the cost of living at the MWHs was high for many women. They spend money for food. Such indirect costs have been a serious challenge for women particularly with low socio-economic status.

*“Life can be easy for those women who have money; they can go and buy from the market. For those women who don’t have money, life is difficult. They pass the night without food. There is no chance.” (*MWH user, FGD)

MWH users mentioned that there were instances in which women pay money for buying gloves and catgut suture from private clinics. Such payments forced some women to discontinue using the MWHs.

*“They also forced us to buy cutgut* suture *from private drug stores. Because of this, I remember one mother who discontinued staying in the MWH.” (*MWH user, FGD)

Health workers mentioned that there have been efforts from the community to reduce the high cost of living in the MWHs by contributing money, maize or coffee. In some MWH catchment areas, the communities have been implementing a motto entitled “one-birr-for one mother”. A health worker illustrated how this has been implemented in the community:

*“There is the so-called ‘one-Birr-for one mother.’ The communities contribute one birr for a pregnant woman. HEWs collect this money from each household. The communities also contribute coffee and maize.”* (Clinician, IDI)

Some health workers also shared their concerns about the sustainability of “one-birr-for one mother” strategy. For instance, they mentioned that there were unnecessary delays in submitting the collected money to the concerned bodies. Some people didn’t have also willingness to contribute money.

*“The communities contribute in-kind and in-cash. However, the contributions are not available on time” (*Clinician, IDI)

*“Recently, we are mobilizing the communities to contribute money. Some of them are willing to give while some of them are not. I doubt the continuity of such community mobilizations and money contributions.”* (Clinician, IDI)

### Information

This theme is crosscutting them that describes about information disseminations and women’s awareness about availability, acceptability and affordability of the MWHs. Health extension workers and clinicians participated in this study mentioned that information about the existence and benefit of MWHs is given both at the health facility and community levels. MWHs user participated in the FGDs also acknowledged the outreach activities and information given by frontline health workers.

*“For me, staying in the MWH is good. It is the HEW who told me to come & wait in the MWH.”* (MWH user, FGD)

*“She visited and advised us to keep household hygiene. She told us not to uncover food utensils after and before feeding; advised us to use our latrine appropriately; informed us to take contraceptives after delivery and advised us not to use blade together.”* (MWH user, FGD)

MWH non-users mentioned that HEWs didn’t inform pregnant women when exactly go to the MWHs. Some HEWs advised pregnant women to go to the MWHs when women feel pain or when labour starts. Such advice from the HEWs has made women not to access the MWH on time. As a result, women may give birth at home or on the way to the health facilities because of sudden labour. A woman who gave birth on the way to the health facilities witnessed:

*“The reason why I didn’t go early is because I was well before labouring started and she (to mean the HEW) informed me to go to the health facilities when I encounter a problem or illness.”* (MWH non-user, IDI)

All non-users mentioned that they are aware of the existence of MWHs in their immediate vicinity and some has started to realize the harmful consequences of home delivery. Nevertheless, health workers mentioned that there were women who didn’t fully understand the aims and benefits of staying in the MWHs.

*“They know the existence of MWHs. However, they don’t know more about the advantage of staying in the MWHs.” (HEW, IDI)*

The aim of MWHs is to reduce complications that may arise during pregnancy by overcoming distance barriers. Complication during pregnancy is uncertain. So, women residing in remote areas need to go to the MWHs before they develop complications. However, the communities perceived that MWHs are constructed for women who develop complications. Some women even considered that women who are going to the MWHs will have an operation.

*“The communities believe that those women who give birth at the health center are weak and couldn’t give birth at home.”* (HEW, IDI)

*“Women don’t go to the MWH because they fear operation. We are happy to give birth naturally (we don’t need an operation). In the clinic, they cut our womb and we feel pain.”* (MWH non-user, IDI)

## Discussions

This qualitative study aimed to provide in-depth insights about the factors influencing women’s access to the MWHs using specific dimensions of access as a guiding framework [19]. As identified by participants, the factors that influence women’s access to the MWHs were both structural and socio-cultural and closely related with dimension of access proposed by Thiede *etal.*

MWHs were viewed as positive by all groups of participants. Both MWH users and non-users know the existence of MWHs in their immediate vicinity and some has recognized the adverse consequences of home delivery practice. In line with this finding, Vian T et al found that women in rural Zambia had good knowledge and all heard about the existence of maternity waiting shelters [26]. Participants agreed that women’s awareness about the existence of MWHs and the adverse consequences of home delivery was due to HEWs health education efforts. Health extension worker’s role as community health educators and promoters of referral linkages in rural Ethiopia was documented elsewhere [13, 27]. Health extension workers are the major sources of information about the location of MWHs; services available at the MWHs and the advantage of staying at the MWHs in rural Ethiopia [13]. However, participants also mentioned that there were women who didn’t recognize the aims and benefits of the MWHs. A former study in rural areas of Ethiopia also showed that more than half of women (51%) did not know the importance of MWHs [13]. Lack of knowledge about the aims of MWHs was cited as one of the reasons for low utilization of MWHs in rural Kenya [8]. Some women believed that MWHs are reserved for weak women and every woman admitted to MWHs will have an operation. The presence of such misperceptions may indicate that women in rural southwest Ethiopia didn’t fully understand the aims of MWHs. In fact, MWHs are designed to prevent but not to treat complications that may arise during pregnancy. Women need to be clearly informed about this to look for the MWHs before they develop complications.

Although the MWHs are believed to serve as a bridge to skilled care by providing temporary shelter near a facility staffed by professionals, long distance, unfavorable roads and lack of transport options remain potential barriers for the accessibility of MWHs in rural southwest Ethiopia. Observers in Sub-Saharan Africa also documented that MWHs didn’t bring services closer to women living in remote areas [28]. Long distance and unavailability of transportations have been potential barriers for utilization of MWHs in low and middle-income countries [9, 12, 14]. This finding suggests that more efforts are expected from the government to facilitate transportation options for women residing in remote areas. Ambulances should be available for women who are marginalized by long distance. In some areas, the roads might be even impassable for ambulances. In this case, a liaison between the already existing social structures like women’s groups and health development armies may facilitate early referral before labour had started at home [27].

However, referring quite a lot of women to the MWHs before their expected date of birth may be difficult due to unavailability of basic facilities at the MWHs and absence of support for their children. Unavailability of basics facilities was seen as potential barrier for most women to accessing the MWHs. Lack of facilities where women and families can stay has been a challenge for the implementation of MWHs in Ethiopia over the last decades [13, 14]. Such unavailability of basic facilities and insufficient classes not only deterred women from accessing MWHs but also eroded health workers motivation to refer many women to the MWH in rural Southwest Ethiopia. The government of Ethiopia had developed and distributed a standardized protocol for managing the MWHs. As clearly stated in the protocol, MWHs need to have infrastructures and basic facilities. Therefore, each MWH are expected to have infrastructures and provide basic facilities including food for MWH users. However, it seems that most of the MWHs in this study area were not constructed based on the standards and lacked the basic facilities. Regular monitoring and evaluation of MWHs may be needed to improve the availability of basic facilities and infrastructures.

One of the basic facilities frequently mentioned by MWH users was unavailability and poor quality of food. Food insecurity while staying at the MWHs was found to be the main challenge in many developing countries implementing MWHs [7, 11, 12, 14]. Women don’t wait at the MWH if food is not available. Available evidence in Ethiopia also confirmed that the majority of women (72.2%) decide to use MWHs if food is available at the MWHs [12]. For most women, the cost of living was high because of unavailability and poor quality of food. Similarly, other studies found that MWH doesn’t ease the financial pressure on services and the high cost of living forced women to discontinue using the MWHs [7, 11, 14].

Reduction of costs associated with using the MWHs and/or subsequent institutional delivery services enable MWHs implementation and utilization more successful [29–31]. Nevertheless, reduction of indirect costs may be tricky for many developing countries, including Ethiopia because of budget deficiencies. For instance, a former study showed that 86% of the MWHs in rural Ethiopia had no budget allocation from government funds in 2016 [13]. There were attempts to cover the indirect costs through community resource mobilization in-kind or in-cash. In fact, this may assist women to bear the financial burdens of staying at the MWHs. Vian T et al also agree that such individual donations or annual community contributions help to support the long-term financial sustainability of maternity shelters [26]. However, in addition to community contributions, programmers also need to look alternative strategies to support the financial sustainability of MWHs.

Similar to other study findings [13, 32], some husbands in this study area facilitated MWH use by accompanying women and bringing foods. However, like many African women [8, 10, 12, 33], women in rural Southwest Ethiopia need their husband’s support and approval to access MWH care services. As limitation of this study, we did not explore the reasons why some husbands did not support and allow women to access the MWHs. In other African country, for instance, in rural Zambia, Sialubanje *et al* found that lack of basic facilities and poor quality of care in the MWHs were the major reasons why the husbands refused women’s referral to the MWHs [33]. In this study area, further studies are needed on why husbands refuse referrals; why they prefer their wives to stay at home until the expected date of delivery and how they could be used as facilitators of referrals.

Finally, the presence of disrespectful care and misalignment between cultural preference and MWH care practices in this study area may suggest poor quality of care. Available evidence showed that poor quality of care has been one of the potential barriers for women to access the MWHs [4, 11]. As this study area has many ethnic groups with different cultures, integrating culturally appropriate care may increase the acceptability of MWHs.

### Strengths and limitations of the study

To improve the trustworthiness of the study findings, we followed different approaches of data collections, including in-depth interviews, FGDs and observations. Some participants were also invited to comment on the research findings and themes. In this study, the views of husbands were not explored because of financial constraints. Efforts were made to explore the husbands’ view through the lens of women, HEWs, and clinicians.

## Conclusions

The factors influencing women’s access to the MWHs were structural and individual and resonate with Thiede et al. dimensions of access. A better understanding of which factors are most influential in preventing women’s access to the MWHs in rural Southwest Ethiopia is needed to appropriately target interventions.

## Supplementary information

**Additional file 1.** ‘A-frame’ of access proposed by Thiede and colleagues.

**Additional file 2.** Checklist to assess the availability of infrastructure and basic facilities at selected MWHs.

**Additional file 3.** Focus group discussion and in-depth interview guidelines.

**Additional file 4.** The Consolidated Criteria for Reporting Qualitative Research checklist: a 32 checklist for interviews and focus groups of participants.

## Data Availability

The dataset used and analyzed during the current study are available from the corresponding author on reasonable request.

## Abbreviation

COREQ: Consolidated criteria for reporting qualitative research
FGDs: Focus group discussions
FMOH: Federal ministry of health
HEWs: Health extension workers
IDIs: In-depth interviews
MWHs: Maternity waiting homes
NGOs: Nongovernmental organizations
SBAs: Skilled birth attendants
