# Supplementary material for: Factors influencing women’s access to the maternity waiting home in rural Southwest Ethiopia: a qualitative exploration
Source: BMC Pregnancy Childbirth. 2020 May 14;20:296. doi: 10.1186/s12884-020-02988-8 (PMC7226938; doi:10.1186/s12884-020-02988-8)
Supplement: Supplementary file 1 — Additional file 1. ‘A-frame’ of access proposed by Thiede and colleagues. [file 12884_2020_2988_MOESM1_ESM.docx]

**Additional file 1**

**Thiede *et al* definition of each dimension of access**

**Availability (physical access):** Refers to the availability of health services at the right time and place. It includes distance between services and residences, transportation options and ability of consumers to reach the health facilities. Availability also includes health provider’s willingness to provide mobile services and undertake mobile services. Furthermore, it implies the type, range, quantity and quality of health services provided.

**Affordability (financial access):** Refers to the fit between the costs of utilization of services and individuals ability to pay

**Acceptability (cultural access):** Refers to the nature of services and how perceived by communities and individuals. This in turn includes attitudes of provider’s vs individual such as sex, age, ethnicity and culture. It also includes provider’s expectation and patient’s expectations.

**Information:** It cuts across all other access dimensions. It includes information and awareness about the availability, acceptability and affordability of health services.
