# Supplementary material for: Factors influencing women’s access to the maternity waiting home in rural Southwest Ethiopia: a qualitative exploration
Source: BMC Pregnancy Childbirth. 2020 May 14;20:296. doi: 10.1186/s12884-020-02988-8 (PMC7226938; doi:10.1186/s12884-020-02988-8)
Supplement: Supplementary file 2 — Additional file 2. Checklist to assess the availability of infrastructure and basic facilities at selected MWHs. [file 12884_2020_2988_MOESM2_ESM.docx]

**Additional file 2**

**Checklist to assess the availability of infrastructure and basic facilities at MWH**

***Name of maternity waiting home: _____________________***

| **Items** | **Yes** | **No** | **Number** |
| --- | --- | --- | --- |
| MWH manual |  |  |  |
| Found inside the health centre |  |  |  |
| Numbers of classes |  |  |  |
| Type of house |  |  |  |
| Kitchen |  |  |  |
| Latrine |  |  |  |
| Water |  |  |  |
| Electricity |  |  |  |
| Shower |  |  |  |
| Television |  |  |  |
| Registration book |  |  |  |
| Have gateway |  |  |  |
| The gateway can pass a car |  |  |  |
| Poster about danger sign |  |  |  |

*Thank you!*
