# Supplementary material for: Factors influencing women’s access to the maternity waiting home in rural Southwest Ethiopia: a qualitative exploration
Source: BMC Pregnancy Childbirth. 2020 May 14;20:296. doi: 10.1186/s12884-020-02988-8 (PMC7226938; doi:10.1186/s12884-020-02988-8)
Supplement: Supplementary file 3 — Additional file 3. Focus group discussion and in-depth interview guidelines. [file 12884_2020_2988_MOESM3_ESM.docx]

**Additional file 3: Focus group discussion and in-depth interview guidelines**

**Focus group discussion guide for users of maternity waiting home**

**Guiding questions organized according to the dimensions of access**

1. How do you know about MWH?

Probe: information provider, outreach activities, and transparency and advantages of MWHs

1. How do you express the acceptance of MWH in your community?

Probe: acceptance by leader, cultural institutions, mothers and families and activities against their culture at the MWHs

1. How did you reach the maternity waiting home?

Probe: the distance, mechanisms of transportation, ups and downs

1. How do you express the direct and indirect cost associated with in reaching and staying at MWH?

Probe: payment for travel, indirect medical cost, cost of basic necessities and communities resource mobilization

1. Once arrived at MWH and in your staying, how do you express the availability of basic facilities and health personals?

Probe: availability of basic necessities, health personals and accommodations and supports

1. How do you express the care given at MWH?

Probe: the intended cares (ANC, counselling, nutrition, family planning)

**In-depth interview guideline for non- users of maternity waiting home**

**Guiding questions organized according to the dimensions of access**

1. Have you heard about MWHs? If so, how do you know?

Probe: information provider, outreach activities, and transparency

1. How do you express the acceptance of MWH in your community?

Probe: acceptance by leader, cultural institutions, mothers and families and activities against their culture at the MWHs

1. If you heard from others, how do you express the direct and indirect cost associated with in reaching and staying at MWH?

Probe: payment for travel, indirect medical cost, cost of basic necessities

1. If you heard from others, how do you express the availability of basic facilities and health personals?

Probe: availability of basic necessities, health personals and accommodations

1. How do you express the direct and indirect cost associated with in reaching and staying at MWH?
2. If you heard from others, how do you express the care given at MWH?

Probe: the intended cares (ANC, counselling, nutrition, family planning)

**In-depth interview guide for health extension workers and clinicians**

***Guiding questions organized according to the dimensions of access***

1. How do you express women and community awareness about MWHs?

Probe: information provider, outreach activities, and transparency

1. How do you express the acceptance of MWH in this community?

Probe: acceptance by leader, cultural institutions, mothers and families and activities against their culture at the MWHs

1. How do women reach at the maternity waiting home?

Probe: the distance, mechanisms of transportation, ups and downs

1. How do you express the direct and indirect cost associated with in reaching and staying at MWH?

Probe: payment for travel, indirect medical cost, cost for basic necessities & communities resource mobilization

1. Once women arrived at MWH and in their staying, how do you express the availability of basic facilities and health personals?

Probe: availability of basic necessities, health personals and accommodations& and supports

1. How do you express the care given at MWH?

Probe: the intended cares (ANC, counselling, nutrition, family planning)
